# Osteonecrosis of the jaw: a rare but possible side effect in thyroid cancer patients treated with tyrosine-kinase inhibitors and bisphosphonates

**DOI:** 10.1007/s40618-021-01634-0

**Published:** 2021-07-21

**Authors:** L. Lorusso, L. Pieruzzi, M. Gabriele, M. Nisi, D. Viola, E. Molinaro, V. Bottici, R. Elisei, L. Agate

**Affiliations:** 1 Endocrinology Unit, Department of Clinical and Experimental Medicine, University of Pisa, Via Paradisa 2, 56124 Pisa, Italy; 2 Department of Surgery, Section of Oral Surgery, University of Pisa, Pisa, Italy

**Keywords:** Thyroid cancer, Sorafenib adverse event, Zoledronic acid, Tyrosine-kinase inhibitors therapy, Osteonecrosis of the jaw

## Abstract

Osteonecrosis of the jaw (ONJ) is a rare but very serious disease that can affect both jaws. It is defined as exposed bone in the maxillofacial region that does not heal within 8 weeks after a health care provider identification. ONJ can occur spontaneously or can be due to drugs like bisphosphonates (BPS) and anti-RANK agents, in patients with no history of external radiation therapy in the craniofacial region. Although in phase 3 trials of tyrosine kinase inhibitors (TKIs) used in thyroid cancer (TC) the ONJ was not reported among the most common side effects, several papers reported the association between ONJ and TKIs, both when they are used alone and in combination with a bisphosphonate. The appearance of an ONJ in a patient with metastatic radio-iodine refractory differentiated TC, treated with zoledronic acid and sorafenib, has put us in front of an important clinical challenge: when a ONJ occurred during TKIs treatment, it really worsens the patients’ quality of life. We should consider that in the case of ONJ a TKI discontinuation becomes necessary, and this could lead to a progression of neoplastic disease. The most important aim of this review is to aware the endocrinologists/oncologists dealing with TC to pay attention to this possible side effect of BPS and TKIs, especially when they are used in association. To significantly reduced the risk of ONJ, both preventive measures before initiating not only antiresorptive therapy but also antiangiogenic agents, and regular dental examinations during the treatment should always be proposed.

## Introduction

Osteonecrosis of the jaw (ONJ) is a rare but very serious disease that can affect both jaws. It is a clinical condition characterized by the presence of one or more bone necrotic lesions that are generally exposed in the oral cavity for at least 8 weeks [1, 2]. This pathology is known since the end of the nineteenth century, when it occurred, along with other side effects, in some workers of matches-making factories after the inhalation of pyrophosphate [3, 4]. Subsequently, the association between ONJ and the use of intravenous bisphosphonate was for the first time reported in 2003 [1], and, since then, several other studies confirmed the association of ONJ, not only with systemic but also with orally administered bisphosphonates (BPS) [1, 2, 5]. In 2007 the American Society for Bone and Mineral Research formulated the definition of BPS-related ONJ (BRONJ) [6], but in 2014, since new drugs able to causing ONJ were identified, the special committee of the American Association of Oral and Maxillofacial Surgeons (AAOMS) [7] suggested to change the name from BRONJ to medication-related ONJ (MRONJ). In fact, drugs like anti-vascular endothelial growth factor (VEGF), anti-RANK agents and tyrosine-kinases inhibitors (TKIs) were demonstrated to be responsible of ONJ too, both when they are used alone or in combination with BPS [8–12].

## ONJ: definition, incidence, and etiology

Recently the International Task Force on Osteonecrosis of the Jaw [13] defined ONJ as exposed bone in the maxillofacial region that does not heal within 8 weeks after identification by a health care provider. MRONJ is the same bone lesion that occurred in patients treated with an antiresorptive agent and/or an anti-angiogenics, and with no history of radiation therapy to the craniofacial region. ONJ/MRONJ can be spontaneous or can follow an alveolar trauma such as dental extraction.

Since oncology patients with bone metastases are exposed to more intensive osteoclast inhibition and to higher BPS doses than those with osteoporosis, the incidence of ONJ is much higher in oncological patients and it ranges from 0 to 12,222 per 100,000 patients-years [14–16]. The prevalence of ONJ in oncological patients treated with intravenous BPS ranges from 0 to 0.186% [17, 18].

Although the pathophysiology of ONJ is not well understood, several hypotheses for explaining the development of this avascular necrosis are available in the literature and these include bone turnover suppression, bone infections, impaired vascularization, immune system malfunction, and oral mucosal damages. This could explain that every drug that can interfere with bone turnover and/or with vascularization, could also increase the risk of ONJ. In particular, regarding BPS, they seem to have a high affinity to hydroxyapatite crystals forming the bone, thereby inhibiting the resorptive action of osteoclasts by induced apoptosis. This effect may indirectly cause a lack of the cytokines released from the osteoclast, prevent the bone healing ability, and may, finally, lead to BRONJ [19]. BPS also seem to have an anti-angiogenic effect. In particular, when a nitrogen-containing BPS (i.e. zoledronic acid) are used, the circulating levels of VEGF, an essential mediator of angiogenesis and of osteogenic differentiation and bone formation, decrease [20]. Since the new TKI therapies also act against the VEGF receptor, interfering with the formation of new blood vessels, and reducing the capability of healing when microtrauma occurs, these drugs may eventually lead to the development of ONJ, and, according to the recent review of the international recommendations, they can be considered as a risk factor on the ONJ development [21].

A synergistic effect of BPS and antiangiogenic agents on the bone was recently described by Christodoulou et al [22]. In this retrospective study, the authors confirmed that the prevalence of ONJ was 1.1% for those oncological patients on intravenous BPS alone and that it increased to 16% when BPS were used in addition to antiangiogenic agents (bevacizumab and sunitinib).

Interestingly, very recently, since FDA warned about the increased risk of ONJ in patients receiving lenvatinib, a pharmacovigilance study, aiming to assess the association between lenvatinib and ONJ and to summarize the clinical features of these cases using the FDA Adverse Events reporting System (FAERS), was published [23]. A total of 9800 lenvatinib-related adverse events were reported and 52 of these (0.5%) were ONJ. This pharmacovigilance study confirmed that the incidence of ONJ due to lenvatinib treatment alone is very low, but it may increase when this treatment is associated to other drugs (i.e. BPS, glucocorticoids) and risk factors (i.e. diabetes and dental procedures).

Regarding the timing of ONJ appearance, according to the study of Jung et al [24], among 1569 incident ONJ cases, 53.3% of ONJ occurred after BPS discontinuation, and most ONJ cases occurred within three years from BPS suspension. In another study [25], it was also demonstrated that MRONJs occur earlier in patients treated concomitantly with TKIs and BPS compared to patients treated with BPS only (after a median exposure of 4.5 and 25.0 months, respectively; *p* = 0.0033).

## Diagnosis of ONJ

According to the AAOMS [7], a diagnosis of MRONJ can be confirmed when patients meet all the following criteria: (1) current or previous treatment with BPS or antiangiogenic agents; (2) exposed bone or bone that can be probed through a fistula, situated within or outside the mouth, in the maxillofacial region that has persisted for longer than 8 weeks; (3) no history of radiation therapy to the jaws or metastatic disease to the jaws.

“Bone that can be probed through a fistula” is a definition referred to those cases (up to one-quarter of patients) that experiences a non-exposed form of MRONJ [26]. To note that the non-exposed form of MRONJ may remain without a correct diagnosis but it would require the same treatment of the classical form of MRONJ.

The most common site of MRONJ is the mandible (less frequently the maxilla), and, in less than 5% of patients, MRONJ is observed in both locations.

Patients with MRONJ may remain asymptomatic for long periods of weeks, months, or even years after the appearance of bone necrosis but most frequently this condition becomes symptomatic with inflammation of surrounding tissues, that can be documented by radiological procedures.

**Fig. 1:**
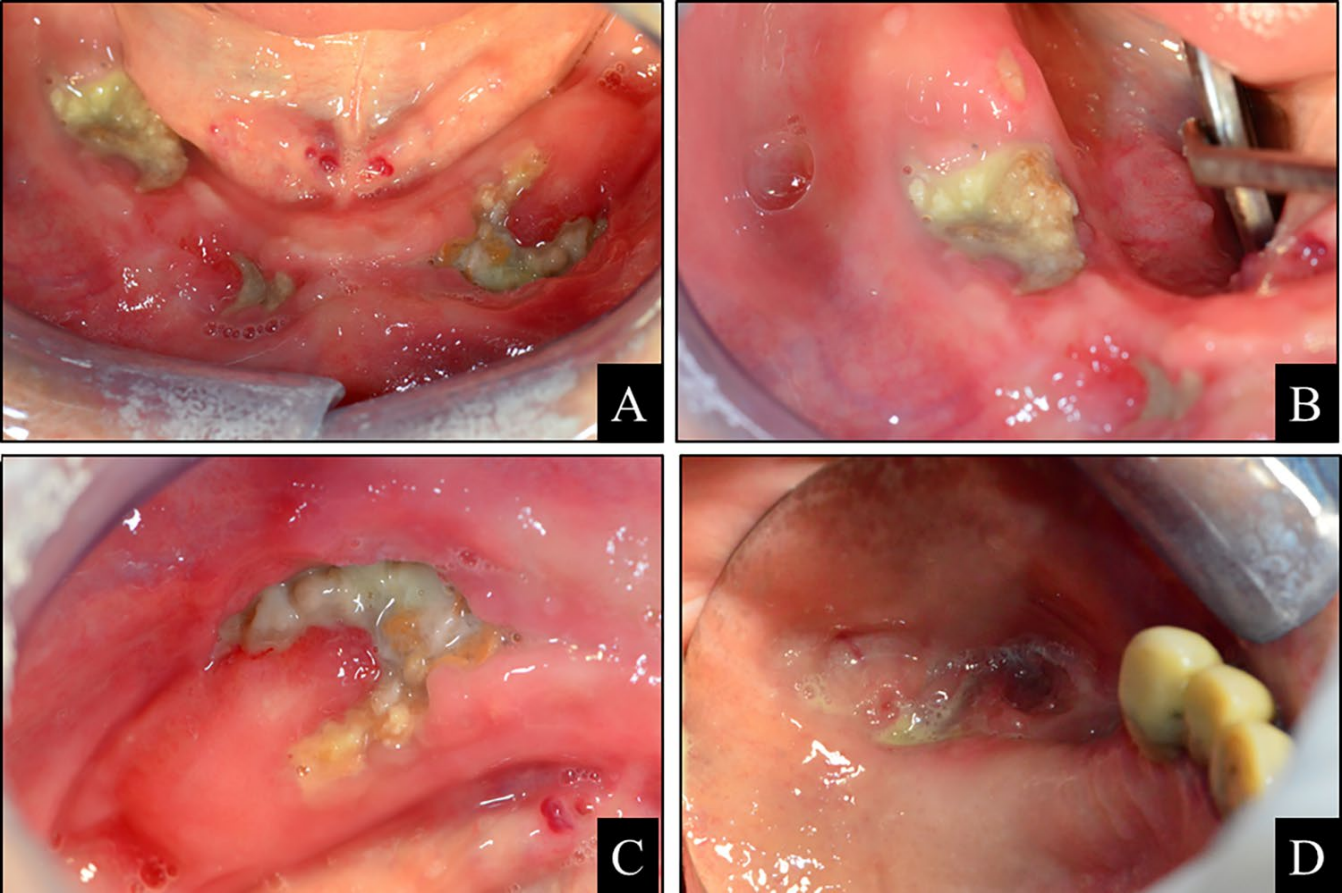
clinical examination of oral cavity showing a large gingival lesion with bone exposure in the mandibular body (**A**), both on the right (**B**) and on the left (**C**), and smaller lesions in the maxilla, bilaterally (**D**): the patient came to our attention complaining bilateral maxillary and mandibular pain with difficulty in feeding

The signs and symptoms of MRONJ are exposed necrotic bone, sinus fistula, difficulties with chewing, eating, and speaking, bad breath, maxillary sinus pain, loose teeth, fracture of the jaw. Figure 1 shows a clinical examination of a mandible and maxilla MRONJ.

AAOMS proposed 5 different clinical stages for MRONJ, from stage 0 to 3rd stages and “at risk” stage [7]. In particular, in stage 0 all those patients with no clinical evidence of necrotic bone but with non-specific symptoms and/or clinical/radiographic findings can be included; stage 1 includes patients with exposed and necrotic bone, or fistulae that probe to bone, who are asymptomatic with no evidence of significant adjacent or regional soft tissue inflammation or infection; stage 2 includes patients with exposed and necrotic bone, or fistulae that probe to bone, associated with infection, as shown by pain and adjacent or regional soft tissue inflammatory swelling, with or without purulent drainage; stage 3 includes patients with exposed and necrotic bone, or fistulae that probe to bone, with evidence of infection, and one or more of the following: (1) pathologic fracture; (2) exposed necrotic bone extending beyond the region of alveolar bone (ie, inferior border and ramus in the mandible, maxillary sinus and zygoma in the maxilla); (3) extra-oral fistula; (4) oral antral/oral nasal communication, (5) osteolysis extending to the inferior border of the mandible or sinus floor. The so-called “at risk stage” includes all those patients who have been treated with BPS/antiangiogenic agents but who have no apparent necrotic bone.

Radiographic criteria are not included for the diagnosis of MRONJ, but, since many studies [27–29] have suggested that the reason for the refractory behavior of MRONJ is that the diagnosis based on bone exposure is too late, an early radiological identification of MRONJ would be ideal. Therefore, some authors focused their attention to identify, by computer tomography scan (CT), some early bone features typical of stage 0 MRONJ. Trabecular bone density seems to be significantly higher in stage 0 MRONJ patients than in the control group or in the at-risk group, especially in the regions close to the alveolar process [30, 31]. In the same way, several studies [31, 32] confirmed that cortical bone thickness, measured by cone-beam CT, is greater in stage 0 patients and early MRONJ patients than in the control and at-risk groups. This means that measuring trabecular bone CT radiodensity values and cortical bone thickness could be simple quantitative methods to radiologically detect the early stages of MRONJ. Except for these features, in advanced-stage disease, there seems to be no significant relationship between stage and imaging features.

## Prevention and therapeutic options of ONJ

The implementation of dental screening and appropriate dental measures before initiating antiresorptive therapy/antiangiogenic agents reduces the risk of ONJ in several prospective studies, especially when compared to patients who did not undergo dental preventive measures [33–35].

Treatment planning should include clinical examination of the oral cavity and a radiographic assessment, and the identification of both acute infection and sites of potential infection to prevent future sequelae that could be exacerbated once drug therapies begin is fundamental.

Individuals receiving monthly intravenous BPS or denosumab for the treatment of oncologic disease have an increased risk of developing ONJ following tooth extraction and thus these procedures should be avoided if possible.

Regarding the treatment of ONJ/MRONJ, in 2014, AAOMS [7] provided the indications for the treatment of patients with ONJ/MRONJ according to the stage of the disease. In particular, at risk-patients, since they have no exposed bone, do not require any treatment, but they should be informed of the risks of developing MRONJ, as well as the signs and symptoms of this disease process.

Stage 0 MROJ requires symptomatic treatment, and conservatively management of other local factors, such as caries and periodontal disease. Systemic management should include medications for chronic pain and control of infection with antibiotics when indicated. Since there is a potential for progression to a higher stage of disease, these patients should be closely monitored.

- Stage 1: patients could benefit from medical management including the use of oral antimicrobial rinses, such as chlorhexidine 0.12%, but no immediate operative treatment is required.
- Stage 2: patients could benefit from the use of both oral antimicrobial rinses in combination with antibiotic therapy. Although local bone and soft tissue infection is not the primary etiology for this condition, the colonization of the exposed bone by sensitive penicillin microbes is a very common occurrence (quinolones, metronidazole, clindamycin, doxycycline, and erythromycin may also be used in those patients who are allergic to penicillin). When systemic antibiotics failure occurred, mainly due to a biofilm formation on the surface of the exposed bone, operative therapy directed at reducing the volume of colonized necrotic bone should be added to antibiotic therapy.
- Stage 3: patients could benefit from debridement, including resection, in combination with antibiotic therapy, which may offer long-term palliation with a resolution of acute infection and pain. Symptomatic patients with stage 3 disease may require resection and immediate reconstruction with a reconstruction plate or an obturator.

Regardless of the disease stage, the mobile bone should be removed to facilitate soft tissue healing. Moreover, the extraction of symptomatic teeth within exposed necrotic bone should be considered since it is unlikely that the extraction will exacerbate the established necrotic process.

## ONJ in thyroid cancer patients treated with TKI

In the last 10 years, for the treatment of thyroid cancer, endocrinologists focused their attention on TKI therapies, small molecules able to inhibit different types of tyrosine kinase receptors, as well as a central mediator of MAPK and PI3K/AKT/mTOR signaling cascades [36]. In particular, sorafenib and lenvatinib were approved for the treatment of differentiated (DTC) and poorly differentiated (PDTC) radioiodine-refractory and progressive thyroid carcinoma [37, 38], while vandetanib and cabozantinib [39, 40] were approved for progressive and metastatic medullary thyroid cancer (MTC). Moreover, a second generation of TKIs have been recently approved for the treatment of thyroid cancer by Food and Drug Administration and are under evaluation by European Medicines Agency (larotrectinib, entrectinib, selpercatinib, and pralsetinib). Although in phase 3 trials of TKI used in thyroid cancer the ONJ was not reported among the most common side effects, as mentioned above, several papers reported the association between ONJ and TKI, both when they are used alone and in combination with BPS [41–45].

Sorafenib is an oral multiple TKI that targets the VEGF receptor family (VEGFR-2 and VEGFR-3) and platelet-derived growth factor receptor family, which play key roles in tumor progression and angiogenesis. It was approved for advanced hepatocellular carcinoma (HCC), for metastatic renal cell cancer (RCC), radioiodine-refractory and progressive DTC and PDTC. Although in DECISION study [37], a phase 3 study that leads to the approval of sorafenib for the treatment of thyroid cancer, the most frequent oral adverse event was mucositis and no cases of ONJ were reported, in the literature, the association between sorafenib and ONJ was reported in several cases. Osteonecrosis has been reported in two HCC patients after few months of sorafenib treatment only, without any history of BPS treatment: in one case it was an ONJ [42], and in the other, a bilateral osteonecrosis of femoral heads was developed [43]. At variance, several are the reports of the occurrence of ONJ during both sorafenib and BPS therapy in RCC patients. According to these studies, this adverse event occurred after an exposure to sorafenib varying from 5 to 36 months and always when sorafenib was used in combination with BPS for bone metastases control [12, 45]. To our knowledge, no cases of MRONJ in thyroid cancer patients treated with sorafenib are present in the literature.

Lenvatinib is another oral TKI with high anti-angiogenic activity, able to inhibit VEGF1-3, FGF1-4, PDGF, KIT, and RET receptors. To date, lenvatinib is approved for the treatment of ^131^I refractory differentiated thyroid cancer, for HCC, and, in combination with everolimus, for RCC. To date, one single case of MRONJ in thyroid cancer patients treated with lenvatinib is described [41]. In particular, Mauceri et al. [41] described a patient with Hürthle cell thyroid cancer, that, since a metastatic progressive disease occurred after initial standard treatment, started a systemic therapy with lenvatinib. After 12 months of lenvatinib intake, the maxillary left first molar was extracted because of deep caries. Healing of the socket was never achieved and, two months after the dental extraction, intraoral examination revealed clinical signs of MRONJ. Treatment with lenvatinib was discontinued by the oncologist and the patient was treated with a combination of ampicillin and sulbactam (1 g intramuscularly twice daily for 7 days) and metronidazole (500 mg orally three times daily for 7 days). Chlorhexidine 0.2% mouthwash (30 ml swished for up to 60 s, three times daily for 14 days) and sodium hyaluronate (local application three times daily for 14 days) were also used. At the latest follow-up, after 6 months, the patient was asymptomatic and the intraoral fistulas had healed.

Cabozantinib is another oral TKI able to inhibit MET, VEGFR2 and RET. It is approved for the treatment of RCC and MTC. Also for cabozantinib a single case of MRONJ occurred in one MTC patient was described [46]. In the paper the authors described the onset of MRONJ in an MTC female patient, enrolled in a double-blind, phase 3 trial, testing the efficacy of orally administered cabozantinib. Three months after the initial intake of cabozantinib, the mandibular left first molar was extracted because of deep caries but no healing of the socket was achieved. Intraoral examination revealed local signs of inflammation and infection, with slight purulent exudation. Orthopantomogram revealed an incomplete bone remodelling of the mandibular left first molar socket and a further assessment by CT scan showed irregularity of the alveolar cortical margin and a sclerotic reaction. Cabozantinib was not discontinued, nor were other prescriptions changed. Since surgical debridement of the socket and antibiotic therapy did not achieve clinical improvement, a segmental ostectomy was performed along with extracting the mandibular left second molar and maintaining antibiotic and antiseptic therapy (oral amoxicillin clavulanate and chlorhexidine 0.2% mouthwash) until mucosal healing had been achieved. Histological assessment of the specimen confirmed the presence of atypical bone necrosis. At a 4‐year follow‐up, the patient remains free of lesions and symptoms.

Very recently, another case of a 61-years old male patient who developed an advanced and unusual case of stage 3 peri-implantitis-induced MRONJ involving the right upper jaw was described by Bennardo et al [47]. For a metastatic RCC, starting from May 2017, the patient received interleukin-2 subcutaneously 3 weeks a month, twice a day for 5 days a week, and then, from June 2017, bevacizumab, every 2 weeks, for 6 months. He also underwent a monthly 4 mg infusion of zoledronic acid for five cycles, which was then replaced, in December 2017, by denosumab every 4 weeks, until September 2019. Moreover, from May to September 2019, he also received nivolumab (10 mg/ml), and then cabozantinib (60 mg). Timing of appearance of ONJ in relation to multidrug therapy was not reported.

To our knowledge to date, no case of MRONJ was described in thyroid cancer patients treated with vandetanib in combination or not with BPS.

Table 1 summarizes the case reports of MRONJ occurred during TKI treatment used in thyroid cancer or in other solid tumors.

**Table 1:** case reports of osteonecrosis of the jaw described in the literature related to tyrosine kinase (TKI) or to TKI and bisphosphonates (BPS)

TKI	Case report	TKI or TKI + BPS	Timing of ONJ appearance after TKI/TKI + BPS inception	Type of treated tumor/disease
Sorafenib	Guillet et al.* [43]	TKI alone	After 10 months	HCC
	Garuti et al. [42]	TKI alone	After 3 months	HCC
Lenvatinib	Mauceri et al. [41]	TKI alone	After 14 months	TC
Cabozantinib	Marino et al. [46]	TKI alone	After 3 months	TC
	Bennardo et al. [47]	Multidrug therapy, including TKI and BPS	Not known	RCC
Axitinib	Patel et al. [50]	TKI alone (nivolumab and pazopanib in the previous years)	After 6 months	RCC
Imatinib	Okubo-Sato et al. [51]	TKI alone	After 10 years	CML
	Gupta et al. [52]	TKI alone	After 2 months	MPN with hypereosinophilia
	Viviano et al. [53]	TKI alone	After 22 months	GIST
Sunitinib	Ashrafi et al. [54]	TKI and BPS	After 5 months from TKI inception After 2 months from BPS inception	RCC
	Agrillo et al. [55]	TKI and BPS (2 cases reported)	Not known	RCC
	Fleissig et al. [56]	TKI alone	Not reported	RCC
	Nicolatou-Galitis et al. [57]	TKI + cisplatinum (case 1) TKI alone (case 2)	Not reported After 18 months	RCC
	Hoefert et al. [58]	TKI and BPS (case 1) TKI and BPS (case 2) TKI and BPS (case 3)	After 6 months After 19 months After 27 months from TKI inception and after 15 months from BPS inception	RCC
	Bozas et al. [59]	TKI and BPS	After 5 months from TKI and 4 days after BPS inception	RCC
	Koch et al. [60]	TKI	After 12 months	RCC
	Brunello et al. [10]	TKI and BPS	After 4 months from TKI inception and after 17 months from BPS inception	RCC
Everolimus	Akkach et al. [61]	TKI	After 6 months	Kidney transplant
	Yamamoto et al. [62]	TKI	After 2 months	Breast cancer
	Omarini et al. [63]	TKI and BPS	After 19 months from TKI inception After 1 month from BPS inception	Breast cancer
	Giancola et al. [64]	TKI and BPS	After 18 months from TKI inception and after 27 months from BPS inception	RCC
	Kim et al. [65]	TKI and BPS	After 3 years from TKI inception and after 6 years of BPS inception	MTC

*CML* chronic myelogenous leukemia, *GIST* gastrointestinal stromal tumors, *HCC* hepatocellular cancer, *MPN* myeloproliferative neoplasm, *RRC* renal cell cancer, *TC* thyroid cancer

*Osteonecrosis of femoral heads

## Our experience with ONJ related to BPS and TKI

A 53-years-old male patient was referred to the Department of Endocrinology, University of Pisa, for a follicular thyroid cancer, with poorly differentiated areas (T3aN0M0). In 1999 he was treated with total thyroidectomy, followed by radioiodine (^131^I) therapy (130 mCi), without subsequent salivary glands symptoms. A remission of the disease was documented after initial treatment.

Since 2003, a progressive thyroglobulin (Tg) increasing levels were observed without any evidence of structural disease.

In 2006, a significant increase of serum thyroglobulin values (from 1.1 ng/ml in 2003 to 50 ng/ml in 2006) associated with local relapse of the disease in thyroid bed and lung micrometastases were documented. The structural disease remained stable until January 2010, when a progression of local disease was documented, and the patient underwent a further surgical treatment followed by neck and mediastinal external radiotherapy for a total amount of 60 Gy. Mandibular and maxillary bones were not involved when external beam radiation on the neck was performed.

Ten months after the last surgery and 8 months after radiotherapy, a further recurrence of local disease in the neck, a numeric and dimensional increase of the lung micrometastases, and the appearance of an osteolytic bone lesion (4.5 cm) of the right clavicle were documented, and, in November 2010, the patient was enrolled in the study protocol “Bayer 14295 Clinical trial on the advance/metastatic radioiodine-refractory DTC (NCT00984282)”. He was assigned to the placebo arm.

In May 2012, about 2 years after enrollment, a significant dimensional progression of the right clavicular lesion with pathological fracture occurred and the appearance of an osteolytic lesion of the left iliac bone was documented. According to protocol indications, the patient entered a cross-over phase, starting 800 mg per day of sorafenib. After 3 months of TKI therapy, based on the presence of bone metastases with local pain, intravenous zoledronic acid therapy was associated with sorafenib treatment, preceded by both an accurate clinical and radiological (orthopantomography) dental examination and the cure of the oral cavity.

For the bone involvement, a local treatment was evaluated and a left iliac lytic lesion percutaneous microwave thermoablation was performed in February 2013. The neoplastic disease remained substantially stable according to the following tbCT scan and to the biochemical examination but, for the appearance of local pain, external radiotherapy was performed on the right clavicular bone lesion in February–March 2013.

In July 2014, 2 years after its inception, the zoledronic acid therapy was permanently discontinued for a dental infection that was completely cured with antibiotic drugs.

In December 2016, about four years after the inception of sorafenib, the patient came to our attention complaining bilateral maxillary and mandibular pain with difficulty in feeding. A clinical examination of the oral cavity showed a large gingival lesion with bone exposure in the mandibular body (Fig. 1, panel A), both on the right (Fig. 1, panel B) and on the left (Fig. 1, panel C), and smaller lesions in the maxilla, bilaterally (Fig. 1, panel D). An odontostomatological consultation diagnosed an extensive aseptic bone necrosis with the appearance of radicular residues and consequently, a surgical removal of those radicular parts and a surgical curettage of the jaw alveolar bone bilaterally were performed.

**Fig. 2:**
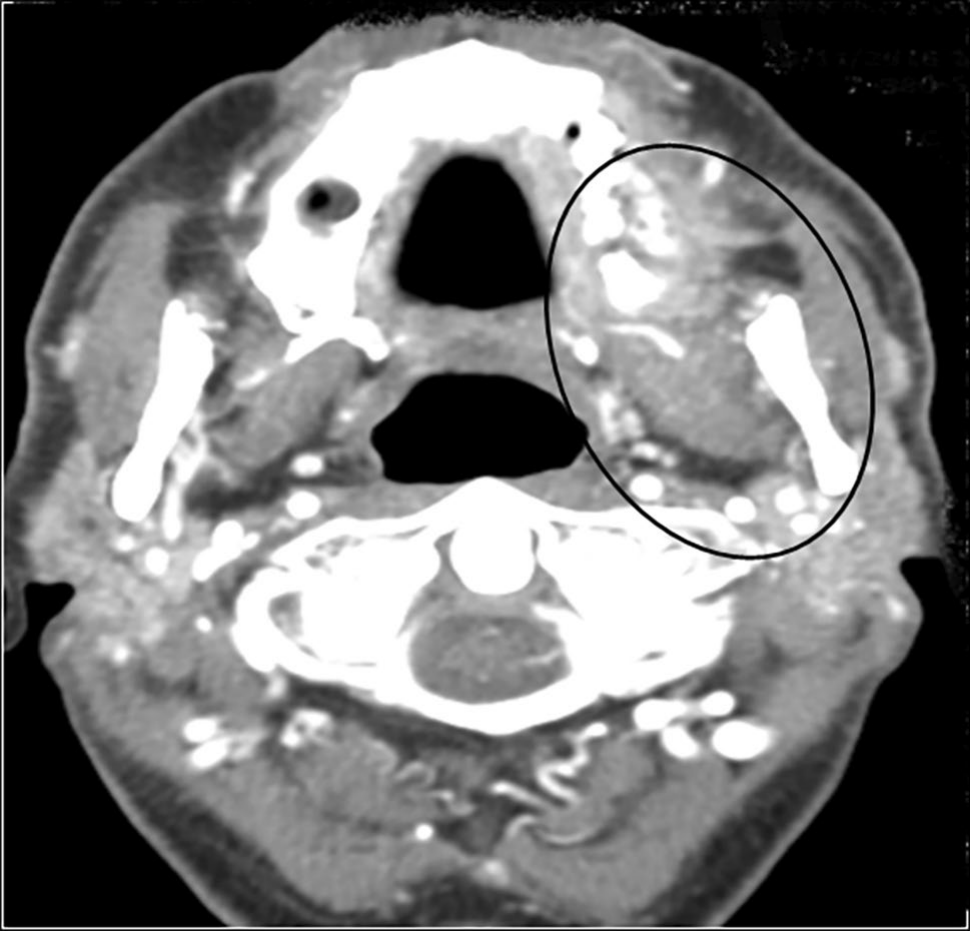
CT scan of the skull image showing an inflammatory morphological pattern of the left maxillary bone with bone erosion, in communication with the oral cavity. The inflammatory lesion involved the contiguous soft tissues too

A CT scan of the skull performed 1 month later, showed an inflammatory morphological pattern of the left maxillary bone, that involved soft tissues, with bone erosion, in communication with the oral cavity (Fig. 2). A further radiological evaluation concluded that maxilla and mandibular bone were both compromised and that the alterations observed were compatible with a ONJ. After a multidisciplinary meeting, including a neuroradiologist, an odontologist, and a maxilla-facial surgeon, sorafenib was discontinued and antibiotic therapy was administered to perform a surgical toilette. In March 2017, the patient underwent surgical removal of a tooth and an osteotomy of the necrotic bone, and a further surgical toilette of maxillary necrotic lesions was performed two months later.

Since the appearance of ONJ sorafenib treatment was discontinued several times to perform surgical toilettes and to let surgical lesions to heal. As per protocol, only when a grade 2 of ONJ adverse event was obtained, sorafenib was started again.

During 2018 and 2019 the patient underwent several radiological procedures and odontostomatological consultations that showed a stable alteration of jaws. Regarding thyroid neoplastic disease, in February 2018 patient underwent a thoracotomy to remove a 6th right rib metastatic lesion and, in May 2019, an embolization and cryoablation of a metastatic bone lesion of the left ilio-pubic branch. A further progression of the disease-induced us to definitively stop sorafenib and start another TKI, lenvatinib, but a worsening of clinical condition occurred and the patient died in April 2020.

## Conclusions

Since, currently, TKI therapy is widely used not only for the treatment of RAI-refractory DTC but for advanced MTC too, and since their association with BPS is frequent when bone metastases are present, endocrinologists/oncologists, dealing with thyroid cancer, should pay attention on this possible side effect due to these drugs, especially when they are used in association.

Metastatic bone disease, as well as in the case we presented, represents a challenging clinical problem in patients with RAI-refractory DTC and metastastic MTC. Since TKIs appear to be less effective in controlling bone metastatic disease in comparison to metastatic disease occurring in other soft tissues (i.e. lungs, liver, and lymph nodes), and a high rate of multiple skeletal-related events following the detection of an initial bone lesion are frequent, according to the current guidelines on DTC management [48], BPS (or denosumab) therapy should be considered in patients with diffuse and/or symptomatic bone metastases from RAI-refractory DTC, either alone or concomitantly with other systemic therapies.

To significantly reduced the risk of ONJ appearance, preventive measures such as oral examinations with appropriate radiographs, oral hygiene instructions, maintenance of good oral health, completion of necessary dental treatments, should always proposed to patients before initiating not only antiresorptive therapy but also antiangiogenic agents and TKIs. Regular dental examinations during the treatment should also encourage earlier treatment of the non-exposed variant of the traditional ONJ.

Unfortunately, when an ONJ occurred during TKI and BPS treatments, it really worsens the quality of life of cancer patients [19, 49], and we should consider that in the case of ONJ it becomes necessary a TKI discontinuation that could lead to a progression of neoplastic disease. A multidisciplinary approach for this kind of patient could be the best practice to avoid an underestimation of both the ONJ, that can have a variable spectrum of presentation and staging, and of the metastatic disease that becomes a high risk of progression if the antineoplastic drug(s) are discontinued.

## Data Availability

Not applicable.

## References

[1] Marx RE (2003). Pamidronate (Aredia) and zoledronate (Zometa) induced avascular necrosis of the jaws: a growing epidemic. J Oral Maxillofac Surg.

[2] Ruggiero SL, Mehrotra B, Rosenberg TJ, Engroff SL (2004). Osteonecrosis of the jaws associated with the use of bisphosphonates: a review of 63 cases. J Oral Maxillofac Surg.

[3] Pollock RA, Brown TW, Rubin DM (2015). “Phossy Jaw” and “Bis-phossy Jaw” of the 19th and the 21st centuries: the diuturnity of John Walker and the friction Match. Craniomaxillofac Trauma Reconstr.

[4] Jacobsen C, Zemann W, Obwegeser JA, Grätz KW, Metzler P (2014). The phosphorous necrosis of the jaws and what can we learn from the past: a comparison of “phossy” and “bisphossy” jaw. Oral Maxillofac Surg.

[5] Otto S, Pautke C, Van den Wyngaert T, Niepel D, Schiødt M (2018). Medication-related osteonecrosis of the jaw: prevention, diagnosis and management in patients with cancer and bone metastases. Cancer Treat Rev.

[6] Khosla S, Burr D, Cauley J, Dempster DW, Ebeling PR, Felsenberg D, Gagel RF, Gilsanz V, Guise T, Koka S (2007). Bisphosphonate-associated osteonecrosis of the jaw: report of a task force of the American Society for Bone and Mineral Research. J Bone Miner Res.

[7] Ruggiero SL, Dodson TB, Fantasia J, Goodday R, Aghaloo T, Mehrotra B, O’Ryan F (2014). American association of oral and maxillofacial surgeons position paper on medication-related osteonecrosis of the jaw—2014 update. J Oral Maxillofac Surg.

[8] Taylor KH, Middlefell LS, Mizen KD (2010). Osteonecrosis of the jaws induced by anti-RANK ligand therapy. Br J Oral Maxillofac Surg.

[9] Van Poznak C (2010). Osteonecrosis of the jaw and bevacizumab therapy. Breast Cancer Res Treat.

[10] Brunello A, Saia G, Bedogni A, Scaglione D, Basso U (2009). Worsening of osteonecrosis of the jaw during treatment with sunitinib in a patient with metastatic renal cell carcinoma. Bone.

[11] Fusco V, Galassi C, Berruti A, Ciuffreda L, Ortega C, Ciccone G, Angeli A, Bertetto O (2011). Osteonecrosis of the jaw after zoledronic acid and denosumab treatment. J Clin Oncol.

[12] Fusco V, Porta C, Saia G, Paglino C, Bettini G, Scoletta M, Bonacina R, Vescovi P, Merigo E, Lo Re G (2015). Osteonecrosis of the jaw in patients with metastatic renal cell cancer treated with bisphosphonates and targeted agents: results of an Italian multicenter study and review of the literature. Clin Genitourin Cancer.

[13] Khan AA, Morrison A, Hanley DA, Felsenberg D, McCauley LK, O’Ryan F, Reid IR, Ruggiero SL, Taguchi A, Tetradis S (2015). Diagnosis and management of osteonecrosis of the jaw: a systematic review and international consensus. J Bone Miner Res.

[14] Khan AA, Rios LP, Sándor GKB, Khan N, Peters E, Rahman MO, Clokie CML, Dore E, Dubois S (2011). Bisphosphonate-associated osteonecrosis of the jaw in Ontario: a survey of oral and maxillofacial surgeons. J Rheumatol.

[15] Tennis P, Rothman KJ, Bohn RL, Tan H, Zavras A, Laskarides C, Calingaert B, Anthony MS (2012). Incidence of osteonecrosis of the jaw among users of bisphosphonates with selected cancers or osteoporosis. Pharmacoepidemiol Drug Saf.

[16] Amadori D, Aglietta M, Alessi B, Gianni L, Ibrahim T, Farina G, Gaion F, Bertoldo F, Santini D, Rondena R (2013). Efficacy and safety of 12-weekly versus 4-weekly zoledronic acid for prolonged treatment of patients with bone metastases from breast cancer (ZOOM): a phase 3, open-label, randomised, non-inferiority trial. Lancet Oncol.

[17] Ganguly S, Divine CL, Aljitawi OS, Abhyankar S, Mcguirk JP, Graves L (2012). Prophylactic use of zoledronic acid to prevent early bone loss is safe and feasible in patients with acute myeloid leukemia undergoing allogeneic stem cell transplantation. Clin Transplant.

[18] Young J, Nickman NA, Biskupiak JE, Barney RB, Gaffney DK, Namjoshi M, Brandt P (2013). Characterization of clinical course and usual care patterns in female metastatic breast cancer patients treated with zoledronic acid. Breast.

[19] Shibahara T (2019). Antiresorptive agent-related osteonecrosis of the jaw (ARONJ): a twist of fate in the bone. Tohoku J Exp Med.

[20] Vincenzi B, Napolitano A, Zoccoli A, Iuliani M, Pantano F, Papapietro N, Denaro V, Santini D, Tonini G (2012). Serum VEGF levels as predictive marker of bisphosphonate-related osteonecrosis of the jaw. J Hematol Oncol.

[21] Khan AA, Morrison A, Kendler DL, Rizzoli R, Hanley DA, Felsenberg D, McCauley LK, O’Ryan F, Reid IR, Ruggiero SL (2017). Case-based review of osteonecrosis of the jaw (ONJ) and application of the international recommendations for management from the international task force on ONJ. J Clin Densitom.

[22] Christodoulou C, Pervena A, Klouvas G, Galani E, Falagas ME, Tsakalos G, Visvikis A, Nikolakopoulou A, Acholos V, Karapanagiotidis G (2009). Combination of bisphosphonates and antiangiogenic factors induces osteonecrosis of the jaw more frequently than bisphosphonates alone. Oncology.

[23] Wenchao L, Qixiang G, Zhuo M, Lihong L, Zhao Z (2021). Lenvatinib and osteonecrosis of the jaw: a pharmacovigilance study. Eur J Cancer.

[24] Jung SY, Suh HS, Park JW, Kwon JW (2019). Drug holiday patterns and bisphosphonate-related osteonecrosis of the jaw. Oral Dis.

[25] van Cann T, Loyson T, Verbiest A, Clement PM, Bechter O, Willems L, Spriet I, Coropciuc R, Politis C, Vandeweyer RO (2018). Incidence of medication-related osteonecrosis of the jaw in patients treated with both bone resorption inhibitors and vascular endothelial growth factor receptor tyrosine kinase inhibitors. Support Care Cancer.

[26] Fedele S, Bedogni G, Scoletta M, Favia G, Colella G, Agrillo A, Bettini G, Di Fede O, Oteri G, Fusco V (2015). Up to a quarter of patients with osteonecrosis of the jaw associated with antiresorptive agents remain undiagnosed. Br J Oral Maxillofac Surg.

[27] Schiodt M, Reibel J, Oturai P, Kofod T (2014). Comparison of nonexposed and exposed bisphosphonate-induced osteonecrosis of the jaws: a retrospective analysis from the Copenhagen cohort and a proposal for an updated classification system. Oral Surg Oral Med Oral Pathol Oral Radiol.

[28] Bagan JV, Hens-Aumente E, Leopoldo-Rodado M, Poveda-Roda R, Bagan L (2012). Bisphosphonate-related osteonecrosis of the jaws: study of the staging system in a series of clinical cases. Oral Oncol.

[29] Devlin H, Greenwall-Cohen J, Benton J, Goodwin TL, Littlewood A, Horner K (2018). Detecting the earliest radiological signs of bisphosphonate-related osteonecrosis. Br Dent J.

[30] Taniguchi T, Ariji Y, Nozawa M, Naitoh M, Kuroiwa Y, Kurita K, Ariji E (2016). Computed tomographic assessment of early changes of the mandible in bisphosphonate-treated patients. Oral Surg Oral Med Oral Pathol Oral Radiol.

[31] Hamada H, Matsuo A, Koizumi T, Satomi T, Chikazu D (2014). A simple evaluation method for early detection of bisphosphonate-related osteonecrosis of the mandible using computed tomography. J Cranio-Maxillofac Surg.

[32] Iwata E, Akashi M, Kishimoto M, Kusumoto J, Hasegawa T, Furudoi S, Komori T (2016). Meaning and limitation of cortical bone width measurement with DentaScan in medication-related osteonecrosis of the jaws. Kobe J Med Sci.

[33] Dimopoulos MA, Kastritis E, Bamia C, Melakopoulos I, Gika D, Roussou M, Migkou M, Eleftherakis-Papaiakovou E, Christoulas D, Terpos E (2009). Reduction of osteonecrosis of the jaw (ONJ) after implementation of preventive measures in patients with multiple myeloma treated with zoledronic acid. Ann Oncol.

[34] Ripamonti CI, Maniezzo M, Campa T, Fagnoni E, Brunelli C, Saibene G, Bareggi C, Ascani L, Cislaghi E (2009). Decreased occurrence of osteonecrosis of the jaw after implementation of dental preventive measures in solid tumour patients with bone metastases treated with bisphosphonates. The experience of the National Cancer Institute of Milan. Ann Oncol.

[35] Bonacina R, Mariani U, Villa F, Villa A (2011). Preventive strategies and clinical implications for bisphosphonate-related osteonecrosis of the Jaw: a review of 282 patients. J Can Dent Assoc.

[36] Xing M (2008). Recent advances in molecular biology of thyroid cancer and their clinical implications. Otolaryngol Clin N Am.

[37] Brose MS, Nutting CM, Jarzab B, Elisei R, Siena S, Bastholt L, De La Fouchardiere C, Pacini F, Paschke R, Shong YK (2014). Sorafenib in radioactive iodine-refractory, locally advanced or metastatic differentiated thyroid cancer: a randomised, double-blind, phase 3 trial. The Lancet.

[38] Schlumberger M, Tahara M, Wirth LJ, Robinson B, Brose MS, Elisei R, Habra MA, Newbold K, Shah MH, Hoff AO (2015). Lenvatinib versus placebo in radioiodine-refractory thyroid cancer. N Engl J Med.

[39] Wells SA, Robinson BG, Gagel RF, Dralle H, Fagin JA, Santoro M, Baudin E, Elisei R, Jarzab B, Vasselli JR (2012). Vandetanib in patients with locally advanced or metastatic medullary thyroid cancer: a randomized, double-blind phase III trial. J Clin Oncol.

[40] Elisei R, Schlumberger MJ, Müller SP, Schöffski P, Brose MS, Shah MH, Licitra L, Jarzab B, Medvedev V, Kreissl MC (2013). Cabozantinib in progressive medullary thyroid cancer. J Clin Oncol.

[41] Mauceri R, Panzarella V, Morreale I, Campisi G (2019). Medication-related osteonecrosis of the jaw in a cancer patient receiving lenvatinib. Int J Oral Maxillofac Surg.

[42] Garuti F, Camelli V, Spinardi L, Bucci L, Trevisani F (2016). Osteonecrosis of the jaw during sorafenib therapy for hepatocellular carcinoma. Tumori.

[43] Guillet M, Walter T, Scoazec JY, Vial T, Lombard-Bohas C, Dumortier J (2010). Sorafenib-induced bilateral osteonecrosis of femoral heads. J Clin Oncol.

[44] Smidt-Hansen T, Folkmar TB, Fode K, Agerbaek M, Donskov F (2013). Combination of zoledronic acid and targeted therapy is active but may induce osteonecrosis of the jaw in patients with metastatic renal cell carcinoma. J Oral Maxillofac Surg.

[45] Beuselinck B, Wolter P, Karadimou A, Elaidi R, Dumez H, Rogiers A, Van Cann T, Willems L, Body JJ, Berkers J (2012). Concomitant oral tyrosine kinase inhibitors and bisphosphonates in advanced renal cell carcinoma with bone metastases. Br J Cancer.

[46] Marino R, Orlandi F, Arecco F, Gandolfo S, Pentenero M (2015). Osteonecrosis of the jaw in a patient receiving cabozantinib. Aust Dent J.

[47] Bennardo F, Buffone C, Muraca D, Antonelli A, Giudice A (2020). Medication-related osteonecrosis of the jaw with spontaneous hemimaxilla exfoliation: report of a case in metastatic renal cancer patient under multidrug therapy. Case Rep Med.

[48] Haugen BR, Alexander EK, Bible KC, Doherty GM, Mandel SJ, Nikiforov YE, Pacini F, Randolph GW, Sawka AM, Schlumberger M (2016). 2015 American Thyroid Association management guidelines for adult patients with thyroid nodules and differentiated thyroid cancer: the American Thyroid Association Guidelines Task Force on thyroid nodules and differentiated thyroid cancer. Thyroid.

[49] Capocci M, Romeo U, Guerra F, Mannocci A, Tenore G, Annibali S, Ottolenghi L (2017). Medication-related osteonecrosis of the jaws (MRONJ) and quality of life evaluation: a pilot study. Clin Ter.

[50] Patel V, Sproat C, Kwok J, Tanna N (2017). Axitinib-related osteonecrosis of the jaw. Oral Surg Oral Med Oral Pathol Oral Radiol.

[51] Okubo-Sato M, Yamagata K, Fukuzawa S, Terada K, Uchida F, Ishibashi-Kanno N, Bukawa H (2021). Medication-related osteonecrosis of the jaw spontaneously occurred in a patient with chronic myelogenous leukemia only by imatinib: a report of a rare case. Case Reports in Dentistry.

[52] Gupta L, Dholam K, Janghel Y, Gurav SV (2021). Osteonecrosis of the jaw associated with imatinib therapy in myeloproliferative neoplasm: a rare case report. Oral Surg Oral Med Oral Pathol Oral Radiol.

[53] Viviano M, Rossi M, Cocca S (2017). A rare case of osteonecrosis of the jaw related to imatinib. J Korean Assoc Oral Maxillofac Surg.

[54] Ashrafi F, Derakhshandeh A, Movahedian B, Moghaddas A (2017). Osteonecrosis of the jaws in patient received bisphosphonates and sunitinib separately: a case report. J Res Pharm Pract.

[55] Agrillo A, Siniscalchi EN, Facchini A, Filiaci F, Ungari C (2012). Osteonecrosis of the jaws in patients assuming bisphosphonates and sunitinib: two case reports. Eur Rev Med Pharmacol Sci.

[56] Fleissig Y, Regev E, Lehman H (2012). Sunitinib related osteonecrosis of jaw: a case report. Oral Surg Oral Med Oral Pathol Oral Radiol.

[57] Nicolatou-Galitis O, Migkou M, Psyrri A, Bamias A, Pectasides D, Economopoulos T, Raber-Durlacher JE, Dimitriadis G, Dimopoulos MA (2012). Gingival bleeding and jaw bone necrosis in patients with metastatic renal cell carcinoma receiving sunitinib: report of 2 cases with clinical implications. Oral Surg Oral Med Oral Pathol Oral Radiol.

[58] Hoefert S, Eufinger H (2010). Sunitinib may raise the risk of bisphosphonate-related osteonecrosis of the jaw: presentation of three cases. Oral Surg Oral Med Oral Pathol Oral Radiol Endodontol.

[59] Bozas G, Roy A, Ramasamy V, Maraveyas A (2010). Osteonecrosis of the jaw after a single bisphosphonate infusion in a patient with metastatic renal cancer treated with sunitinib. Onkologie.

[60] Koch FP, Walter C, Hansen T, Jäger E, Wagner W (2011). Osteonecrosis of the jaw related to sunitinib. Oral Maxillofac Surg.

[61] Akkach S, Shukla L, Morgan D (2019). Everolimus-induced osteonecrosis of the jaw in the absence of bisphosphonates: a case report. Br J Oral Maxillofac Surg.

[62] Yamamoto D, Tsubota Y, Utsunomiya T, Sueoka N, Ueda A, Endo K, Yoshikawa K, Kon M (2017). Osteonecrosis of the jaw associated with everolimus: a case report. Mol Clin Oncol.

[63] Omarini C, Filieri ME, Depenni R, Grizzi G, Cascinu S, Piacentini F (2017). Osteonecrosis of the jaw in a breast cancer patient treated with everolimus and a single dose of zoledronic acid. Breast J.

[64] Giancola F, Campisi G, Lo-Russo L, Muzio LL, Di Fede O (2013). Osteonecrosis of the jaw related to everolimus and bisphosphonate: a unique case report?. Ann Stomatol (Roma).

[65] Kim DW, Jung YS, Park HS, Jung HD (2013). Osteonecrosis of the jaw related to everolimus: a case report. Br J Oral Maxillofac Surg.

